# A Causal Role for V5/MT Neurons Coding Motion-Disparity Conjunctions in Resolving Perceptual Ambiguity

**DOI:** 10.1016/j.cub.2013.06.023

**Published:** 2013-08-05

**Authors:** Kristine Krug, Nela Cicmil, Andrew J. Parker, Bruce G. Cumming

**Affiliations:** 1 Department of Physiology, Anatomy, and Genetics, Oxford University, Sherrington Building, Oxford OX1 3PT, UK; 2 Laboratory of Sensorimotor Research, National Eye Institute, National Institutes of Health, Bethesda, MD 20892-4435, USA

## Abstract

Judgments about the perceptual appearance of visual objects require the combination of multiple parameters, like location, direction, color, speed, and depth. Our understanding of perceptual judgments has been greatly informed by studies of ambiguous figures, which take on different appearances depending upon the brain state of the observer. Here we probe the neural mechanisms hypothesized as responsible for judging the apparent direction of rotation of ambiguous structure from motion (SFM) stimuli. Resolving the rotation direction of SFM cylinders requires the conjoint decoding of direction of motion and binocular depth signals [1, 2]. Within cortical visual area V5/MT of two macaque monkeys, we applied electrical stimulation at sites with consistent multiunit tuning to combinations of binocular depth and direction of motion, while the monkey made perceptual decisions about the rotation of SFM stimuli. For both ambiguous and unambiguous SFM figures, rotation judgments shifted as if we had added a specific conjunction of disparity and motion signals to the stimulus elements. This is the first causal demonstration that the activity of neurons in V5/MT contributes directly to the perception of SFM stimuli and by implication to decoding the specific conjunction of disparity and motion, the two different visual cues whose combination drives the perceptual judgment.

## Highlights

• Targeted search for brain sites encoding conjunctions of stereo and motion
• Electrical microstimulation alters perceptual choice and resolves visual ambiguity
• Causal evidence for direct role of V5/MT in cue combination
• Stimulation at cortical sites with conjoint encoding is particularly effective

## Graphical Abstract

**Figure:**
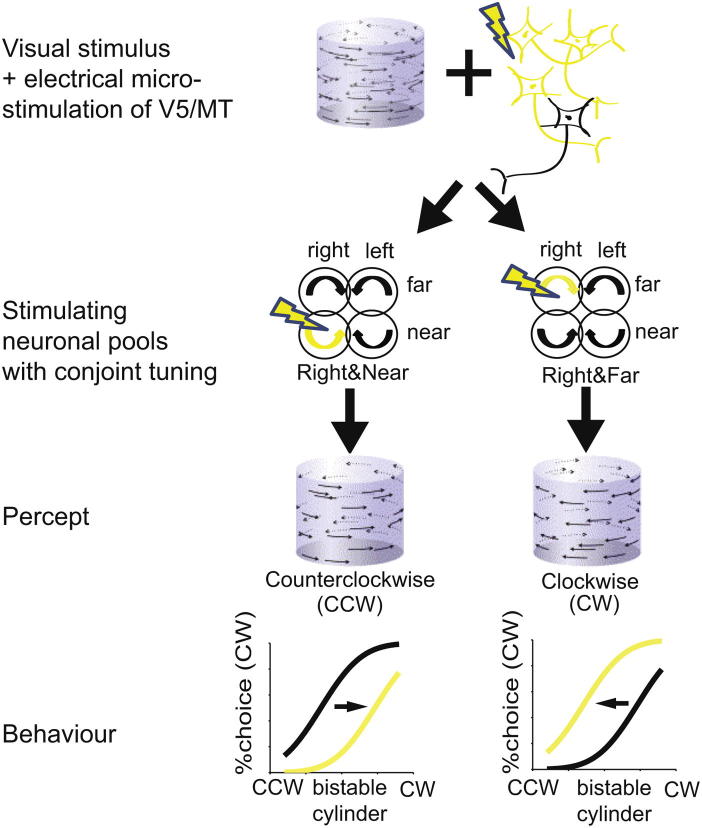

## Results

**Figure 1:**
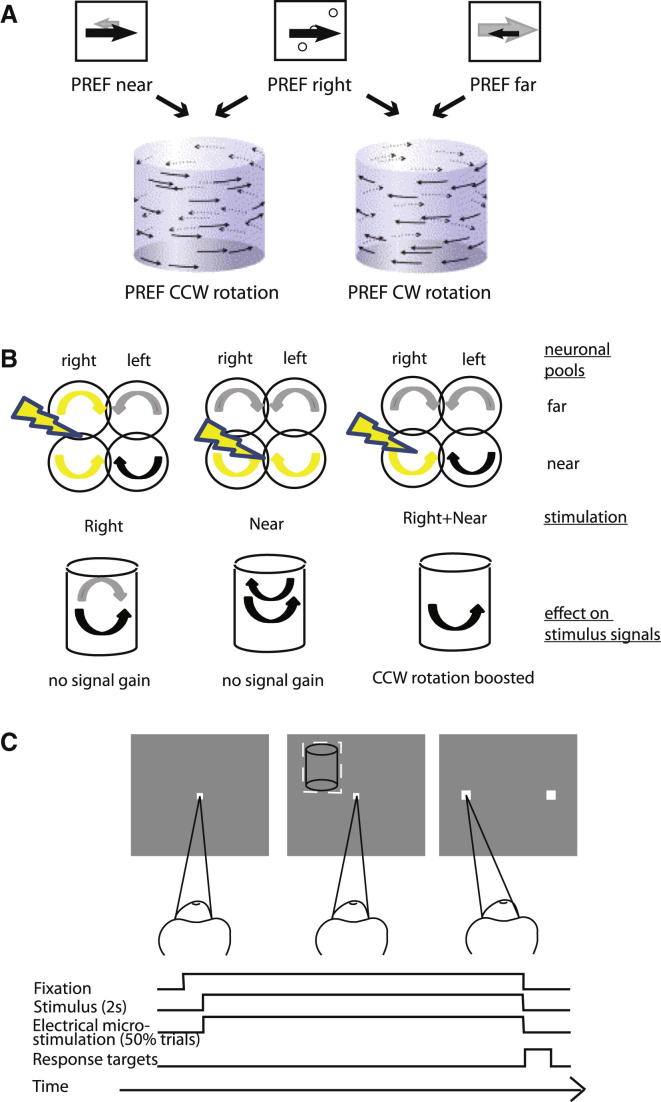
Stimulus and Task (A) The preference (PREF) for a direction of SFM cylinder rotation depends on the specific combination of preferences for binocular disparity and direction of motion. The SFM cylinder stimulus was made up of two transparent sheets of random dots moving in opposite directions, so the direction of rotation was disambiguated by addition of binocular disparities of the same magnitude but opposite sign to the two sets of random dots (see also Movie S1 for an illustration of the stimulus). A neuronal preference for rightward motion alone cannot define a preference for rotation of the cylinder, as both rightward and leftward motion are present in the cylinder stimulus. If rightward motion is preferred in the near-depth plane, CCW rotation would be preferred by the neuron, but if rightward motion is preferred in the far-depth plane, the neuron would be selective for CW rotation. (B) Schematic diagram of different neuronal pools in visual area V5/MT and the expected effect of microstimulation on SFM cylinder perception. For instance, if electrical microstimulation of V5/MT neurons injects a pure rightward motion signal that affects the appearance of features at all disparities, such a signal should not alter the reported rotation direction of a SFM cylinder. Similarly, boosting of neuronal signals for near disparities independent of motion direction would not render a specific rotation percept more likely. Injection of both a rightward motion signal (independent of disparity) and a near disparity signal (independent of motion) will also not change the rotation direction. For stimulation to change the reported direction of rotation, the injected electrical signal must differentially affect the activity of neurons jointly encoding combinations of motion and binocular depth. (C) During a typical trial, the fixation point appeared first. Once the fixation point was acquired by the animal, the cylinder stimulus would appear in the multiunit receptive field, characterized previously. On a random 50% of trials, electrical microstimulation was applied in visual area V5/MT during stimulus presentation. After a 2 s presentation time, cylinder and fixation point disappeared, and two choice targets were presented. The choice targets would either appear to the left and right of where the fixation point had been (monkey FLE) or to either side of the fixation point along the motion axis of the cylinder in this experiment (monkey ICA). To receive a fluid reward, the animal had to saccade to the correct choice target. For the ambiguous cylinder, choices were rewarded 50% of the time at random. Breaking fixation before the fixation point was turned off would lead to the trial being aborted and a brief timeout. See also Movie S1.

Neurons in V5/MT are selective for motion direction and binocular disparity [3–5]. When these visual parameters are examined separately, recording and electrical microstimulation experiments show that V5/MT neurons selective for these parameters make causal contributions to perceptual signals for motion [6–8] and binocular depth [9–11]. Rotating structure from motion (SFM) cylinders comprise two transparent surfaces of random dots, which move in opposite directions; assignment of dots with opposite motion directions to different visual depth surfaces defines rotation direction (Figures 1A and 1B). While V5/MT neurons can be tuned to both a motion direction and a binocular depth [9], it is only at the level of area MST that motion preference reverses for near and far disparities, which would allow a more complete representation of the stimulus [12]. Using electrical microstimulation, we tested whether V5/MT neurons causally contribute to judgments about this stimulus, which is resolved by the specific conjunction of motion and depth information. If neurons in V5/MT are not read out in this task specifically with reference to their conjoint coding, this microstimulation is expected to have no systematic effect on perceived rotation.

**Figure 2:**
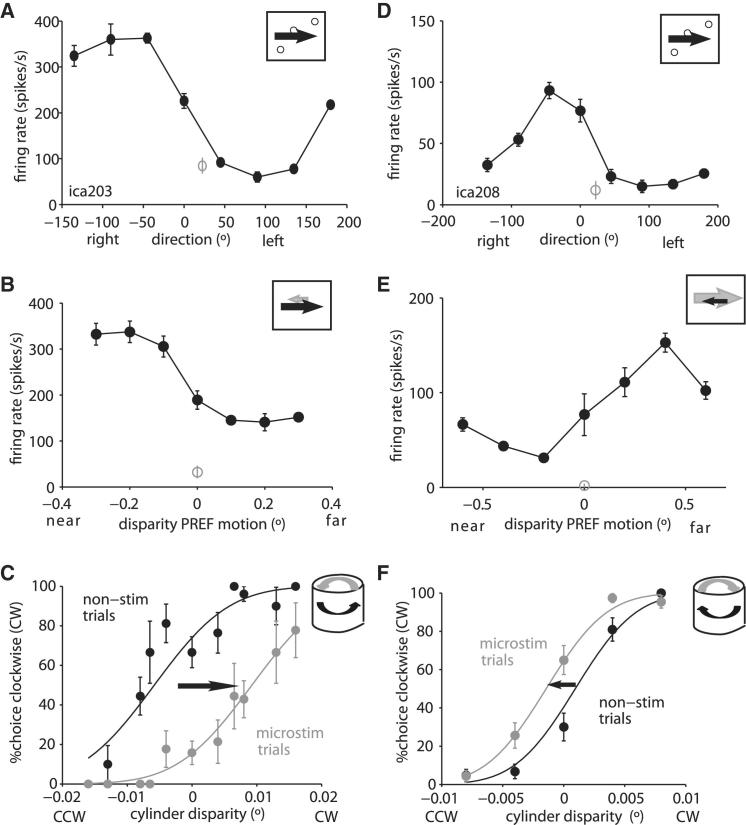
MUA Tuning and Behavioral Shifts due to Microstimulation for Two Example Sites (A and B) At the first example site (ica203), MUA activity is selective for (A) rightward motion and (B) near disparity. (C) Electrical microstimulation at this site causes the appearance of CCW cylinder rotation. The horizontal shift in the psychometric function due to electrical microstimulation is equivalent to addition of 0.015° of binocular disparity to the cylinder stimulus. (D and E) In the same monkey, another site (ica208) is again selective for (D) rightward motion, but this time also for (E) far disparity. (F) Electrical stimulation of this site resulted again in more choices in the direction predicted by neuronal selectivity: more choices CW over a wide range of cylinder disparities. The signal is equivalent to a shift of 0.002°. Error bars depict the SEM. See also Figure S1 and, for another two examples from the second monkey, Figure S2.

Two monkeys were trained to discriminate the direction of rotation of a SFM cylinder (defined by the added binocular disparity) with an eye movement response (Figure 1C). The monkeys worked at close to the psychophysical threshold for binocular disparity. After the electrode entered V5/MT, we recorded multiunit activity (MUA) every 100 μm and searched for sites that showed consistent tuning to both the same direction of motion and the same binocular disparity over a 300 μm stretch of cortex (see also Figure S1 available online). We retracted the electrode to the middle of an identified stretch and quantitatively assessed tuning for direction of motion and for binocular disparity in the cylinder stimulus (Figures 2A, 2B, 2D, and 2E). Cylinder stimuli were matched in position, size, direction of motion, and speed to the preferences of neurons at the chosen site. While the animals judged the direction of rotation on each trial, we applied electrical microstimulation on 50% of those trials, pseudorandomly interleaved with nonstimulated trials for comparison. Animals received fluid reward for correct responses to the visual stimulus, regardless of microstimulation. Data were analyzed from 48 cortical sites with significant MUA tuning to cylinder disparity (ANOVA, p < 0.05) in two monkeys (see the Supplemental Experimental Procedures).

### Effect of Electrical Stimulation on Cylinder Discrimination

We hypothesized that stimulation at a cortical site where MUA is tuned to rightward motion and near disparity should boost the rightward motion signal at the front surface of the cylinder: the animal should consequently choose counterclockwise (CCW) rotation more often (Figures 1A and 1B). Figures 2A–2C show such a site tuned to CCW rotation: across all cylinder disparities, stimulation of this cortical site increased the proportion of CCW choices. The electrical signal seemed to be integrated with the neural signals evoked by the visual stimulation across the range of tested disparities. This is not simply due to boosting choices favoring the rightward motion direction. In the same monkey, we stimulated another site with MUA preference for rightward motion but in conjunction with far disparity; this time, we significantly increased the choices of clockwise (CW) rotation (Figures 2D–2F). Neither disparity selectivity alone nor direction selectivity alone can explain this pattern of results (see also Figure S2).

**Figure 3:**
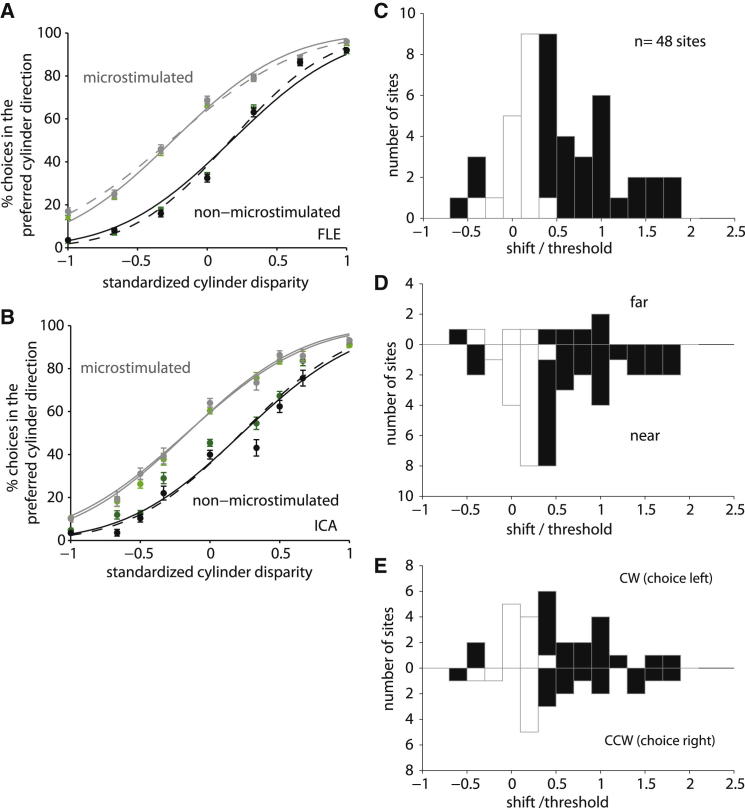
Summary of the Microstimulation Effect (A) This figure illustrates the shifts when we fitted the standardized psychophysical data pooled across microstimulation sites in monkey FLE. The range of cylinder disparities was standardized for all 20 microstimulation sites, such that the largest disparities at each site were set equal to −1 and 1. Data were averaged across sites. The proportion of choices in the preferred direction was plotted against the standardized cylinder disparity, and pairs of cumulative Gaussians were fitted. Data from all sites are shown in green; data for sites with a significant shift are shown in black and gray. For the significant stimulation sites, one pair of fits, for microstimulated (in gray) and nonmicrostimulated trials (in black), was only allowed to differ in their horizontal offset (solid line); the other pair in offset and slope (1/SD) of the fitted curve (dashed line). For FLE, the pooled shift measured +0.42 and the SD (which is a measure of psychometric threshold) was 0.64. This yields a ratio of shift/SD of 0.66, equivalent to almost two thirds of threshold. The pooled data for all microstimulation sites (green) shows an almost identical pattern (shift/SD = 0.62). The difference is small because most sites showed a significant shift and there were two significant shifts in the nonpreferred direction. Error bars depict the SEM. (B) For monkey ICA, response data were standardized and pooled for microstimulation sites with significant shifts (in black and gray) and across all sites (in green), as in (A). The pooled shift for significant microstimulation sites measured +0.39 and the SD 0.66. The shift is equivalent to about two thirds of threshold (SD), with a ratio of shift/SD 0.59 (for all sites shift/SD = 0.36). (C) The distribution of normalized microstimulation shifts across all 48 sites from both monkeys is shown. The majority of significant horizontal shifts with electrical microstimulation (black bars; χ^2^, p < 0.05) are in the direction predicted by the preference at the stimulated site (shift/threshold > 0), with only three significant shifts in the null direction. Some shifts of the psychometric curve are larger than discrimination threshold (SD) (shift/threshold > 1). (D) Normalized shifts plotted separately for sites with a preference for far and near disparities. While there were overall fewer sites with a preference for far disparity, there seemed to be little difference in the distribution. (E) Normalized shifts were plotted separately for sites with a preference for CW or CCW response preference. No significant differences between the distributions of normalized shifts were observed for all comparisons (χ^2^, p > 0.05). See also Figure S3.

We calculated the effect of electrical microstimulation as if it were an additional component of the visual stimulation (see the Supplemental Experimental Procedures). Across all sites in both animals, we fitted the psychometric functions with pairs of cumulative Gaussians that were allowed to differ only in their mean (see Figures 2C and 2F) and measured the horizontal shift between the positions of the 50% points between the two curves. Stimulation sites were assigned positive shifts if the monkey’s choices were shifted toward the neural preference, as determined by the MUA tuning for cylinder stimuli; negative shifts were in the opposite direction. In one monkey (ICA), the mean shift was equivalent to addition of 0.010° (SD ± 0.038) of binocular disparity to the stimulus across 28 sites; in the other monkey (FLE), the mean shift was 0.020° (SD ± 0.029) across 20 sites. We assessed the significance of each measured shift by fitting a single cumulative Gaussian to the combined data at a single site and comparing log likelihoods (χ^2^, p < 0.05). For ICA, 16 out of 28 sites showed a significant shift with a mean of 0.017°; for FLE, 15 out of 20 sites were individually significant with a mean of 0.026°. For both monkeys, most shifts were positive and therefore in the direction predicted by the cylinder preference at the microstimulation site (Figures 3A and 3B).

To compare stimulation effects across stimulation sites and monkeys regardless of stimulus eccentricity and psychometric threshold, we normalized the magnitude of the stimulation effect by dividing the horizontal shift by the psychometric threshold at each site. This “normalized shift” is conceptually equivalent to the use of “normalized equivalent deviations” [13, 14]. Again, positive shifts represent sites where the effect of electrical microstimulation increases perceptual reports in favor of the neurons’ cylinder rotation preference. Across all 48 cortical sites, the median normalized shift is 0.38 (significantly different from 0, Wilcoxon sign rank, p < 0.0001; Figure 3C) and did not differ significantly between monkeys (Wilcoxon rank sum, p = 0.19; see also Figure S3).

Both near and far disparity sites have significant shifts (Figure 3D), with median normalized shifts not significantly different (far = 0.32; near = 0.39; Wilcoxon rank sum, p = 0.40). Consistent with previous reports [4, 5, 15, 16], in V5/MT we identified fewer sites (9/48) at which neurons were tuned to far disparities. Nonetheless, for sites with a particular preference for motion direction, microstimulation had opposite effects on SFM judgments depending on the disparity selectivity of the site. Our results cannot be explained by an effect of microstimulation only on direction judgments, even if combined with some strategy such as only reporting the motion of the near surface. When we plotted normalized shifts, broken down by preferred response direction, we identified significant shifts for both CW (target left) and CCW choices (target right) (Figure 3E). The median, normalized shifts for CW rotation (0.38, n = 28) and for CCW rotation (0.37, n = 20) did not significantly differ (Wilcoxon rank sum, p = 0.70). The distributions of normalized shifts were in both cases not significantly different from each other (χ^2^ test, p > 0.05). The measured shifts with microstimulation could not be explained by changes in vergence or the use of paired rather than independent Gaussian fits (see the Supplemental Experimental Procedures; see Figures 3A and 3B for a comparison of different Gaussian fits).

### Comparison of Microstimulation in the Cylinder and a Planar Disparity Task

DeAngelis et al. [11] showed that electrical microstimulation of disparity-tuned sites in V5/MT influenced perceptual decisions in a depth-discrimination task, based on altering the proportion of dots that carried a binocular disparity signal. From their study, means and slopes for individually fitted sigmoid curves using logistic regression were available. We refitted our data with the same logistic regression used by those authors for direct comparison between the two studies. All results continue to be expressed as normalized shifts as defined above, but change of the fitted model, particularly the use of independent sigmoid curves for stimulated and nonstimulated cases, alters the detailed numerical values in comparison with the previous section.

**Figure 4:**
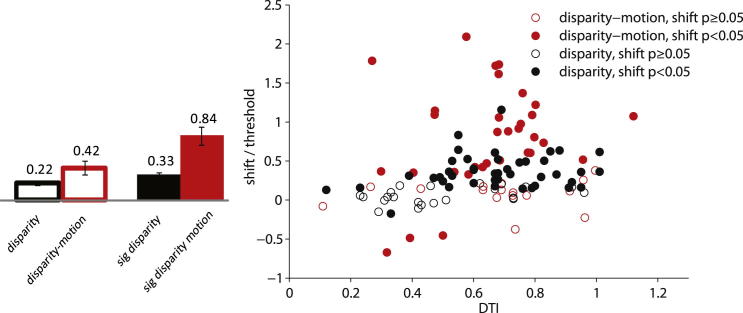
Comparison with V5/MT Microstimulation in a Planar Depth Task Psychometric data from this study were fitted with logistic regression [8] (red filled and unfilled circles) and compared to logistic regression fits to microstimulation data for a disparity discrimination task [11] (black filled and unfilled circles). The horizontal shifts were normalized by the threshold and plotted against the disparity-tuning index (DTI). While the mean DTI between the two studies did not differ (t test, p = 0.33), the normalized horizontal shift induced with microstimulation in our combined disparity-motion task (median 0.42 SD ± 0.63, n = 48) was larger than for DeAngelis et al.’s [11] disparity task (median 0.22 SD ± 0.24, n = 65; Wilcoxon rank sum, p < 0.01). Medians and SEM are shown in the bar graph. To exclude differences in site selectivity, we also compared only microstimulation sites with a significant shift. Again mean DTIs were comparable (t test, p = 0.56), but normalized shifts were considerably larger when microstimulation was applied during our task depending on the specific conjunction of disparity and motion (our data, median 0.84, SD ± 0.64, n = 32; DeAngelis et al., median 0.33, SD ± 0.22, n = 43; Wilcoxon rank sum p < 0.0001). p values in the figure key are for significant shifts induced by electrical stimulation (filled circles).

For disparity discrimination, normalized shift due to microstimulation is correlated with the disparity tuning index (DTI) obtained from MUA at the stimulated site [11]. We also calculated the DTI for our MUA data. The mean DTIs found in the two studies were not significantly different (DeAngelis et al., 0.62, n = 65; this study, 0.66, n = 48; t test, p = 0.33). However, in our data set, only one monkey showed a significant correlation between DTI and normalized shift (combined data, Spearman’s r = 0.14, p = 0.33; FLE, r = 0.45, p < 0.05, n = 20; ICA, r = 0.07, p = 0.74, n = 28; Figure 4). On visual inspection, our data set had fewer sites with very low DTI and nonsignificant shifts. One reason could be that we applied more-stringent selection criteria for inclusion of a cortical site, which constrained the range of DTIs in our data set. When we examined the relationship between DTI and the logistic regression shifts by animal using an analysis of covariance in our data set (n = 48), the slopes for the two monkeys did not differ significantly (p = 0.14), but there was a main effect of animal (p = 0.03). This was not the case for sites with significant shifts induced by microstimulation (p = 0.25, n = 32).

Across the range of DTI, we find systematically larger effects of microstimulation in our task—a median normalized logistic shift of 0.42 (SD ± 0.63, n = 48) in our data compared with 0.22 (SD ± 0.24, n = 65) for DeAngelis et al.’s (Wilcoxon rank sum, p < 0.01; Figure 4). The same holds if we restrict the comparison to sites with significant shifts (0.84 ± 0.64, n = 32, versus 0.33 ± 0.22, n = 43; Wilcoxon rank sum, p < 0.0001). Electrical microstimulation of V5/MT seems to have a stronger effect in our task that depended upon the conjunction of motion and disparity cues than a task based on disparity alone. This reinforces the view that the microstimulation effects we report result from a specialized functional organization of V5/MT for the relationship between disparity and motion, rather than as a byproduct of separate compartments in V5/MT, each compartment contributing separately to disparity and motion perception.

## Discussion

Electrical microstimulation of V5/MT biased choices in the direction expected from local neuronal selectivity for a specific combination of two different visual cues. We demonstrate that electrical signals in V5/MT can causally influence the perceived conjunction of disparity and motion, and thus alter the perception of SFM figures.

Previous experiments established that electrical microstimulation in area V5/MT can influence the perceived direction of motion or the reported binocular depth separately [7, 11]. However, the effect on SFM cylinders cannot result from simply boosting neuronal signals for either left or right motion direction alone without reference to binocular disparity: this would boost signals in the near and far surfaces without any consistent effect upon cylinder rotation. Our data confirm this specificity: the effect of stimulation reverses sign with disparity preference. We therefore reasoned that stimulation at sites with no disparity selectivity would be less informative. We did not stimulate at such locations, and so we cannot exclude the possibility of a small effect at such sites (e.g., if these signals were attributed to near surfaces by default). Any such effect, however, cannot explain the reversal with disparity preference.

The requirement for decoding conjunctions may explain why the effect of microstimulation here is larger than for disparity discrimination alone [11]. V5/MT contains some neurons that are not selective for the conjunction of motion and binocular disparity and other neurons that are. Therefore, the number of neurons potentially available for our task is smaller and more specific than those available for a simple disparity task. Provided that the electrode is located carefully, this means that the signal injected by microstimulation drives a greater fraction of the available neurons, so the psychophysical effect is larger. Although great care was taken to ensure that the stimulation parameters and the procedure for choosing stimulation sites were closely similar between the two studies, it is always possible that other subtle differences contribute. Regardless, the difference in size of microstimulation effect between the two studies warrants further study.

This argument also presupposes that neurons selective to the conjunction are clustered together in V5/MT, as suggested by our MUA recordings. The idea that that clustering is related to the strength of the electrical microstimulation effect is supported by a recent study in extrastriate visual area MSTd on multisensory heading perception [17]. Early combined microstimulation and recording experiments in motor cortex estimated that the effective spread of a 20μA current would be about 100–150μm [18]. These dimensions would be consistent with stimulating neurons within a direction column and a patch of similar disparity preference [11, 19, 20]. Horizontal connectivity within V5/MT is clustered at approximately 2 mm intervals [21], consistent with connecting neurons of similar direction and disparity preference. If the spread of microstimulation is largely determined by the architecture of local circuitry, our results suggest that these local connections reflect the conjunction of disparity and motion signals.

DeAngelis and Newsome [22] studied a motion discrimination task, in which the visual stimulus was matched carefully to both the disparity and motion selectivity of the V5/MT receptive field: in two out of three monkeys, microstimulation effects for motion were stronger at sites that were less well tuned to disparity. However, when there was a microstimulation effect at a disparity selective site, it was generally stronger when the motion stimulus was presented at the disparity preferred at that site. Also in V5/MT, Sasaki and Uka [23] showed that the correlation of neuronal activity with perceptual choice was similar for discrimination of either binocular disparity or direction of motion. These various results are not inconsistent with conjoint coding for motion and disparity, but in both tasks, the other stimulus component could be (and was at least partially) discounted. In contrast, the perceptual decisions about SFM stimuli in our task required the decoding of specific conjunctions of motion and depth signals, rather than about the visual motion or depth parameter in isolation.

Consistent with the results presented here, larger correlations between single neuron firing and perceptual choice (choice probability) have been found in V5/MT for discriminating the direction of rotation of the SFM cylinder stimulus, as used in this study, compared to motion discrimination or depth discrimination tasks alone [6, 9, 10]. Larger choice probabilities have been proposed to indicate a closer link with perception [24], but the link between choice probability and contribution to the perceptual task is not straightforward [25]. One way to reconcile these studies is to suggest that when an animal’s task depends on only a single perceptual attribute, V5/MT neurons with conjoint encoding properties may be involved in a perceptual decision, but these neurons are “read out” in a way that is capable of ignoring the coding for conjunction.

A readout from neurons conjointly coding disparity and motion, as found here for V5/MT, is required as a substrate for a number of psychophysical interactions between motion and stereo, including the depth-dependent motion aftereffect [26]. Neurophysiological recordings in V5/MT previously correlated neuronal responses to resolving perceptual ambiguity for bistable percepts like SFM and binocular rivalry [9, 27], but the level of direct contribution of such signals to perceptual decisions has been debated [28, 29]. For example, greater numbers of temporal and prefrontal neurons appear to follow perceptual dominance in other visual tasks [30, 31]. Our results suggest that these responses related to perceptual dominance might be causally driven by perceptual signals located in visual areas at earlier stages of processing.

In conclusion, our results show a strong causal link between activity in extrastriate visual area V5/MT and perception of SFM stimuli. V5/MT appears to have a specific and robust contribution to perceptual decisions about complex visual objects defined by conjunctions of motion and binocular depth. The coding of perceptual conjunctions within neural circuits may be a general encoding strategy within extrastriate cortex for cues that are commonly encountered in combination in the visual environment.

## Supplemental Information

Document S1. Supplemental Experimental Procedures and Figures S1–S3

Movie S1. Ambiguous, Rotating SFM Cylinder, Related to Figure 1 Illustration of the type of stimulus used in this study. Consistent with an orthographic projection of a three-dimensional cylinder, the peak velocity of the stimulus dots was at the midline of cylinder image; i.e., the dot velocity increased as they approached the midline and then decreased as they moved toward the lateral edges of the cylinder. The cylinder stimulus depicted here is presented at zero binocular disparity, and therefore its direction of rotation is ambiguous. While the percept at a given time is that of coherent rotation, the perceived direction will switch at intervals. For the 2 s presentation in our study, the stimulus should not switch, but in roughly half of the trials, the stimulus would be perceived as rotating CW, and in the other half as CCW. The cylinder stimulus is disambiguated by application of different binocular disparities to leftward- and the rightward-moving dots.
