# Supplementary material for: A Causal Role for V5/MT Neurons Coding Motion-Disparity Conjunctions in Resolving Perceptual Ambiguity
Source: Curr Biol. 2013 Aug 5;23(15):1454–9. doi: 10.1016/j.cub.2013.06.023 (PMC3739008; doi:10.1016/j.cub.2013.06.023)
Supplement: Document S1. Supplemental Experimental Procedures and Figures S1–S3 [file mmc1.pdf]

Current Biology, Volume *23*

Supplemental Information

A Causal Role for V5/MT Neurons Coding Motion-Disparity Conjunctions in Resolving Perceptual Ambiguity

Kristine Krug, Nela Cicmil, Andrew J. Parker, and Bruce G. Cumming

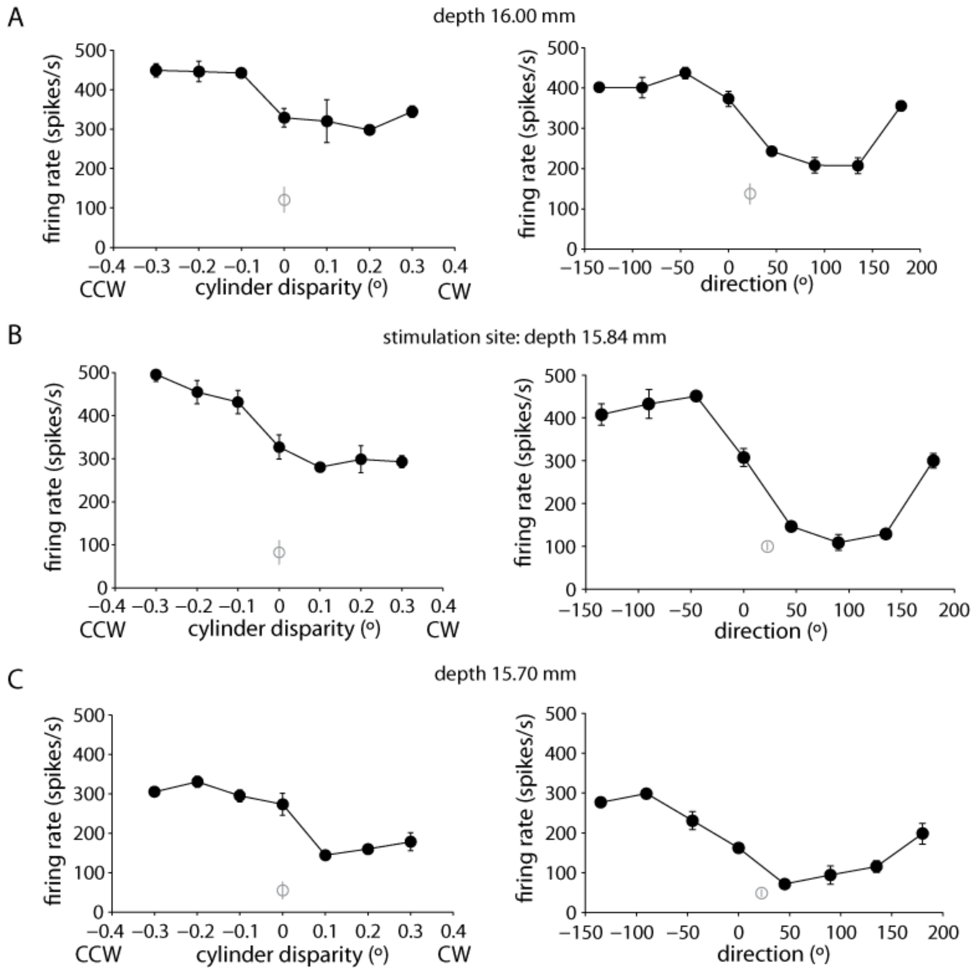

Figure S1. V5/MT Sequence of direction and cylinder rotation tuning around one stimulation site, related to Figure 2

Sequence of cylinder disparity and direction of motion tuning for one microstimulation site shown in Figure 2 (ica203). Sites for electrical microstimulation were selected, based on consistent tuning for cylinder disparity (left) and motion (right). The multi-unit activity (MUA) at this site showed a consistent preference for CCW rotation at a depth of **(A)** 16 mm, **(B)** 15.84 mm – the stimulation site in this experiment, and **(C)** at 15.70 mm from the first entry into cortex. Filled circles represent the mean MUA firing rate, open circles represent responses to blank. Error bars depict ±1 SEM.

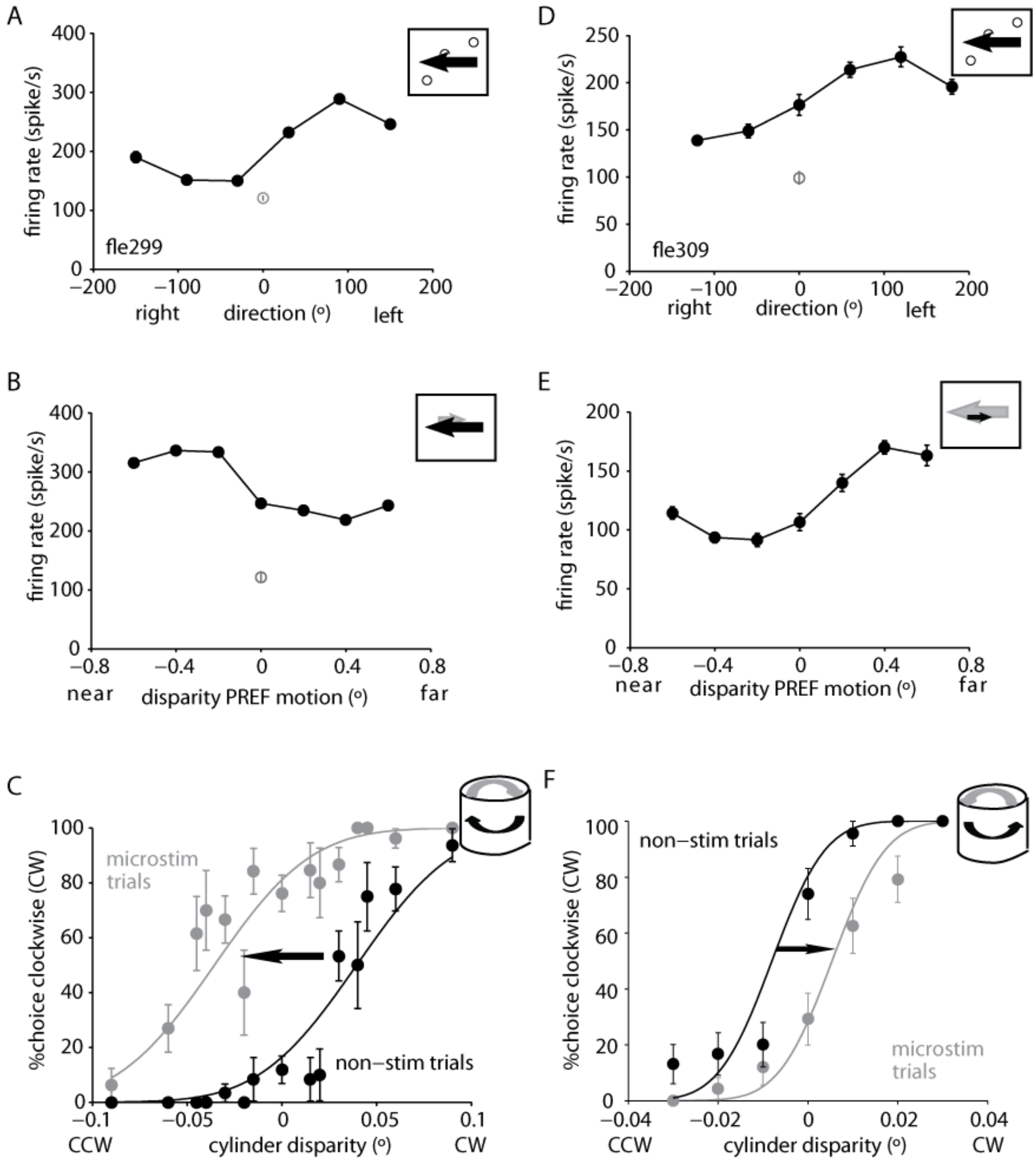

Figure S2. Tuning and microstimulation at two example microstimulation sites, related to Figure 2
MUA tuning and behavioral shifts due to microstimulation for two example sites from the second monkey FLE (two sites from ICA are presented in Figure 2). At this site (fle299), MUA activity is selective for **(A)** leftwards motion and **(B)** near disparity. Error bars depict SEM. **(C)** Electrical microstimulation at this site causes CW cylinder rotation. The horizontal shift in the psychometric function due to electrical microstimulation is equivalent to adding 0.075° of binocular disparity to the cylinder stimulus. In the same monkey, another site (fle309) is again selective for **(D)** leftwards motion, but this time also for **(E)** far disparity. **(F)** Stimulating this site electrically again resulted in more choices in the direction predicted by neuronal selectivity: more choices CCW over a wide range of cylinder disparities. The electrical microstimulation effect is equivalent to an added disparity of 0.014°.

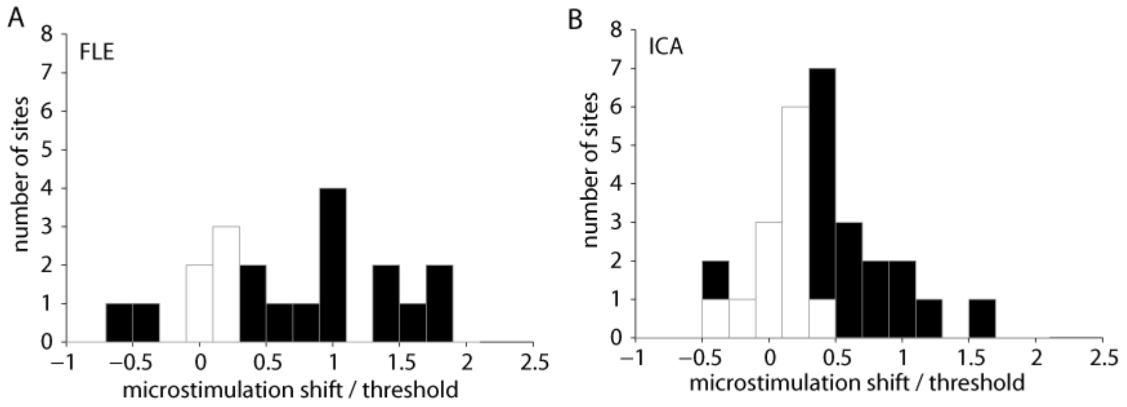

Figure S3. Normalized shift distribution for each monkey, related to Figure 3

The data shown in Figure 3C are presented here separately for each monkey. **(A)** The distribution of normalized microstimulation shifts across all 20 microstimulation sites of monkey FLE. **(B)** The distribution of normalized microstimulation shifts across all 28 sites of monkey ICA. The median normalized shifts for the two monkeys were at 0.64 (FLE, n=20) and 0.35 (ICA, n=28) not significantly different from each other (Wilcoxon ranksum, p=0.19), but they were both significantly different from zero (Wilcoxon sign rank, FLE p<0.01, ICA p<0.001). The shift (in degrees) at each site was normalized by the threshold (SD) of the fitted Gaussian to compare across sites and tasks. The majority of significant shifts (black bars; χ^2, $p<0.05$) were in the positive direction predicted by the preference at the stimulated site (shift/threshold > 0).

Supplemental Experimental Procedures

Animals
Two Rhesus monkeys (Macaca mulatta) were used at two research institutions (monkey FLE at Oxford University, UK; monkey ICA at the NEI/NIH in Bethesda, USA). Procedures were closely similar for the two sets of experiments; any differences in set-up or procedures are provided below. Animals were implanted under general anesthesia with a head holder, scleral search coils, and a chamber for access to the cortex. For monkey FLE, all procedures conformed to UK Home Office Regulations. For monkey ICA, all procedures were in agreement with the Public Health Service policy on the humane care and use of laboratory animals and the Institute Animal Care and Use Committee of the NEI approved all protocols.

Visual stimuli
Different images were presented to each of the two eyes in a stereoscope. In Oxford (FLE), a Wheatstone stereoscope set-up consisted of two EIZO Flexscan monitors with a mean luminance of 42 cd/m^2 and a refresh rate of 72 Hz. Viewing distance was 84 cm, screen size 21° x 17° and pixel size 0.0163°. At the NIH (ICA), two DLP projectors were used (Project Design Ev02sx+) with crossed, linearly polarized filters in front of each eye and projected on a single screen (Stewart Filmscreen 150). The crosstalk between the images was 0.5%. The mean luminance of the display viewed through polarizing filter was 17.5 cd/m^2 and the refresh rate was 60 Hz. Viewing distance was 156 cm, screen size 28° x 19° and pixel size 0.019°.

The SFM cylinder stimuli consisted of two transparent surfaces of random dots moving in opposite directions with a sinusoidal velocity profile (see also Movie S1). The random dots were black and white squares in front of a mid-grey background. Dot size was either 0.2° x 0.2° (FLE) or 0.18° x 0.18° (ICA); dot density for both animals was 25%. A fraction of dots was removed and replotted at a new random location on each frame (FLE: 2% of dots; ICA: 1%). At zero binocular disparity, the direction of rotation of the cylinder is bistable: at times, it is perceived as rotating clockwise (CW), at others counterclockwise (CCW) (conventions as viewed from above). The direction of rotation can be disambiguated by applying different binocular disparities to the two sets of dots moving in opposite directions. For instance, when leftwards-moving dots are moved nearer and the rightwards-moving dots further away, the direction of rotation of the cylinder becomes unambiguously CW (Figure 1A).

Behaviour
After fixation training, both animals were initially trained to discriminate the direction of SFM stimuli rotating about a vertical axis near the fixation point, indicating their response with an eye movement to one of two choice targets. When they did this reliably near disparity threshold, the rotation axis of SFM stimuli was changed away from vertical. Finally, the animals were trained to discriminate SFM stimuli at different positions across the visual hemifield contralateral from the chamber placement. Recording commenced when animals were operating at disparity threshold over the tested range of eccentricities and orientations. Across the stimulation sites in this study, psychometric thresholds differed between the two monkeys (ICA median 0.006°, SD ±0.045°, n=28; FLE median 0.013°, SD ±0.016°, n=20; t-test on log values, $p<0.01$). Therefore, when comparing or pooling data across the two monkeys, where necessary, we normalized by the psychometric threshold obtained at the respective site.

Experimental Protocol
During stimulus presentation the animal had to maintain fixation (window diameter 1°-2°) on the fixation spot on the screen. Recordings and electrical micro-stimulation were carried out with single tungsten electrodes with polyimide tubing (MicroProbe Inc; Impedance 0.1-0.3 MΩ). Extrastriate visual area V5/MT was approached posteriorly through primary and secondary visual cortex. We

identified the cortical area through (i) the known sequence of grey- and white matter on approach, (ii) neuronal preference for direction of motion and binocular disparity [1-2], (iii) clustering of neurons of similar direction and disparity preferences [3-4], (iv) known retinotopic map of V5/MT and (iv) the known relationship between eccentricity and receptive field size [5]. Once we entered V5/MT, multi-unit activity was assessed in 100 µm steps. First, the visual field location of the multi-unit (MU) receptive field was identified; then the selectivity to direction of motion was quantitatively determined with a patch of dots with 100% motion coherence (i.e. on a given trial, all dots moved in the same direction). The MUA receptive field position was confirmed with a patch of dots moving in the preferred direction. Then, we assessed disparity selectivity with a SFM cylinder matched to receptive field size and motion preferences (i.e. one direction of motion in the preferred direction, the other in the opposite [null] direction for this site). We carried out 44 electrode penetrations. We mapped in total 554 V5/MT sites, of which 419 (76%) were tuned to both disparity and direction of motion, 89 (16%) were tuned to motion direction alone and 40 (7%) failed to exhibit clear direction tuning. 6 sites (1%) showed inconsistent tuning for a single unit at this site and the MUA recorded in the neighborhood.

The aim was to identify cortical sites with 300 µm of consistent tuning for cylinder direction of rotation. When such a site had been identified, the electrode was withdrawn to the center of that cortical stretch. The cylinder stimulus was then carefully matched to the MUA receptive field position, size, direction and speed preference. The direction tuning and cylinder disparity tuning were quantitatively measured and stored to the computer's hard-disk memory. Then, the animal discriminated the direction of cylinder rotation in a two-alternative forced-choice task with a fixed duration of 2 s (Figure 1C). Direction of rotation was either in the preferred or the null direction for this site. The stimulus was presented at a minimum of five binocular disparities, which separated the front and back surfaces of the cylinder. On a randomly selected 50% of the trials, we electrically stimulated the site with a 20 µA biphasic pulse (200 µs cathodal stimulation followed 200 µs anodal stimulation) at 200 Hz, whilst the monkey viewed the cylinder rotation. Both monkeys were rewarded with a drop of fluid for making a correct choice about the visual stimulus presented. They were neither trained nor rewarded to detect the microstimulation signal[6]. For zero disparity cylinders, animals were rewarded on 50% of the trials, which were randomly selected.

Analysis

Only cortical sites with at least 10 microstimulated trials and 10 non-microstimulated trials for at least 5 different disparities were included in the analysis. To be included, sites had also to show significant MUA tuning to cylinder disparity (ANOVA $p<0.05$). The exception is one site from FLE, for which cylinder tuning had not been recorded to hard disk, but which had been assessed as disparity tuned based on the tuning curve plotted online during the experiment.

To assess how electrical microstimulation affected behavioral responses, we plotted the proportion of reports in one direction as a function of cylinder disparity for stimulated and non-stimulated trials separately (the psychometric functions). Using Matlab command *fminsearch,* these two sets of data were fitted (with a maximum likelihood estimate) with a pair of cumulative Gaussians which were only allowed to differ in the mean μ:

$$P_{CW} = \frac{1}{2}\left(1 + erf\left[\frac{x - \mu}{\sigma\sqrt{2}}\right]\right)$$

where μ is the mean of the distribution, σ is the standard deviation, and *erf* is the error function. P_{CW} corresponds to the probability of making a CW choice. The shift is the horizontal offset between the two Gaussian curves fitted to the microstimulated and the non-microstimulated trials and was measured in degrees ($\mu_1 - \mu_2$). A shift was deemed significant when the data were significantly better described by such a pair of Gaussians rather than when fit with a single cumulative Gaussian

(comparison of log-likelihoods; χ^2-statistic with 1 d.f.). In order to obtain a measure of shift that was independent of the x-axis units, we divided the shift by the SD of the fitted Gaussian (the threshold).

We defined MUA by counting the number of events where measured voltage exceeded a defined threshold. Because these events were stored, the threshold could be adjusted offline to yield consistent levels of MUA. Typically, the peak response in direction and disparity tuning curves was set to 200-300 impulses/s. Disparity-tuning curves were fitted with a cubic spline interpolation. The Disparity Tuning Index (DTI) was defined as:

$$DTI = 1- (R_{min} - S)/(R_{max} - S)$$

where R_{max} is the maximum fitted response, R_{min} is the minimum fitted response, and S denotes spontaneous activity (response to a uniform grey screen). For ICA, these blank stimuli were collected interleaved with the disparity stimuli, for FLE during an immediately preceding block of directional stimuli. Large values of DTI (around 1) correspond to strong disparity tuning, and values near zero correspond to weak tuning.

We compared the size of the stimulation effect in our experiment to a study [7] that assessed the effects of electrical microstimulation on monkeys' judgments of a near or far binocularly correlated disparity target amidst noise. This study fitted behavioral responses with sigmoid curves using logistic regression [8] :

$$P = \frac{1}{1 + \exp[-(\beta_0 + (\beta_1 \cdot stim) + (\beta_2 \cdot corr) + (\beta_3 \cdot stim \cdot corr))]}$$

where P refers to the probability of making a preferred decision, *stim* reflects the presence or absence of microstimulation, *corr* contains the binocular correlation level of the stimulus. For comparison, we also fit our responses with the same logistic regression. The horizontal shift of the psychometric function due to electrical microstimulation is calculated as β_1/β_2 [8]. Because the two studies use different visual parameters, we compare shifts expressed as a fraction of threshold (defined as the stimulus strength producing 84.1% correct responses).

Vergence

Microstimulation in disparity selective areas might conceivably have caused changes in the state of binocular vergence. Because this would have affected the absolute disparity of both surfaces of the cylinder, not change the depth order between the two opposite motion surfaces of the stimulus, it should not affect the perceived direction of rotation. Nonetheless, to ensure that electrical stimulation did not affect perceptual report through some idiosyncratic effect of changes in binocular vergence, we compared vergence between electrically stimulated and non-stimulated trials for monkey FLE, which had two eye coils in place. While we found a small but statistically significant difference of -0.0039° (paired t-test, $p < 0.05$, n=20), if this small change in vergence were related to electrical microstimulation, we would expect to see opposite effects at sites with a near disparity preference and a far disparity preference. However, there is no significant difference in mean vergence between microstimulation sites with a near disparity preference or a far disparity preference (unpaired t-test, p=0.45, confidence interval -0.0103, 0.0047). Microstimulation shift in the PREF cylinder rotation direction was neither correlated with the mean vergence difference between stimulated and non-stimulated sites (Pearson's r = -0.13, p = 0.58, n=20) nor with the absolute size of the vergence difference (-0.01, p = 0.96, n=20).

Fitting of paired cumulative Gaussians

For the measurement of normalized shifts, we used paired cumulative Gaussians that were only allowed to differ in their means not in their standard deviation (SD), which determines the slope of the cumulative Gaussian. This procedure assumes that electrical microstimulation does not substantially degrade psychophysical performance on the electrically stimulated trials. For perceptual judgments of motion and binocular depth in V5/MT, this assumption is valid [7-8]. Using the pooled data, we tested this assumption for conjoint encoding of motion and binocular depth, by comparing the fits of the paired model assuming the same SD for both curves to fitting two cumulative Gaussians of different means *and* SD (see also main paper Figure 3A, 3B – solid versus dashed lines).

For both monkeys, allowing the fitted curves to differ in their SD did significantly improve the fit for significant microstimulation sites (Figure 3B: ICA; χ^2, p=0.04; Figure 3A: FLE; χ^2, p<0.0001). In the unpaired model for monkey FLE, the SD for the non-microstimulated trials was lower than for the microstimulated trials (Figure 3A, dashed lines: 0.56 and 0.73 respectively), but this difference did not contribute to the measurement of the shift between stimulated and non-stimulated trials (Figure 3A; 0.42 for the paired model—solid lines; 0.43 for the unpaired model—dashed lines). This was also the case for monkey ICA (Figure 3B, dashed lines: SD 0.62, 0.69; shift paired: 0.39, shift unpaired: 0.39). Although the differences in the slope of the fitted functions are small, they are consistent with the idea that the electrical microstimulation may have been mildly detrimental one some trials for choice performance of one of the two animals. However, the main effect of microstimulation remains the shift in mean position of the cumulative Gaussian.

Supplemental References

1. Zeki, S.M. (1974). Functional organization of a visual area in the posterior bank of the superior temporal sulcus of the rhesus monkey. J. Physiol. *236*, 549-573.

2. Maunsell, J.H.R., and van Essen, D.C. (1983). Functional properties of neurons in middle temporal visual area of the macaque monkey. II. Binocular interactions and sensitivity to binocular disparity. J. Neurophysiol. *49*, 1148-1166.

3. Albright, T.D., Desimone, R., and Gross, C.G. (1984). Columnar organization of directionally selective cells in visual area MT of the macaque. J. Neurophysiol. *51*, 16-31.

4. DeAngelis, G.C., and Newsome, W.T. (1999). Organization of disparity-selective neurons in macaque area MT. J. Neurosci. *19*, 1398-1415.

5. Albright, T.D., and Desimone, R. (1987). Local precision of visuotopic organization in the middle temporal area (MT) of the macaque. Exp. Brain. Res. *65*, 582-592.

6. Ni, A.M., and Maunsell, J.H. (2010). Microstimulation reveals limits in detecting different signals from a local cortical region. Curr. Biol. *20*, 824-828.

7. DeAngelis, G.C., Cumming, B.G., and Newsome, W.T. (1998). Cortical area MT and the perception of stereoscopic depth. Nature *394*, 677-680.

8. Salzman, C.D., Murasugi, C.M., Britten, K.H., and Newsome, W.T. (1992). Microstimulation in Visual Area MT: Effects on Direction Discrimination Performance. J. Neurosci. *12*, 2331-2355.
