# Supplementary figures and images for: A Causal Role for V5/MT Neurons Coding Motion-Disparity Conjunctions in Resolving Perceptual Ambiguity
Source: Curr Biol. 2013 Aug 5;23(15):1454–9. doi: 10.1016/j.cub.2013.06.023 (PMC3739008; doi:10.1016/j.cub.2013.06.023)

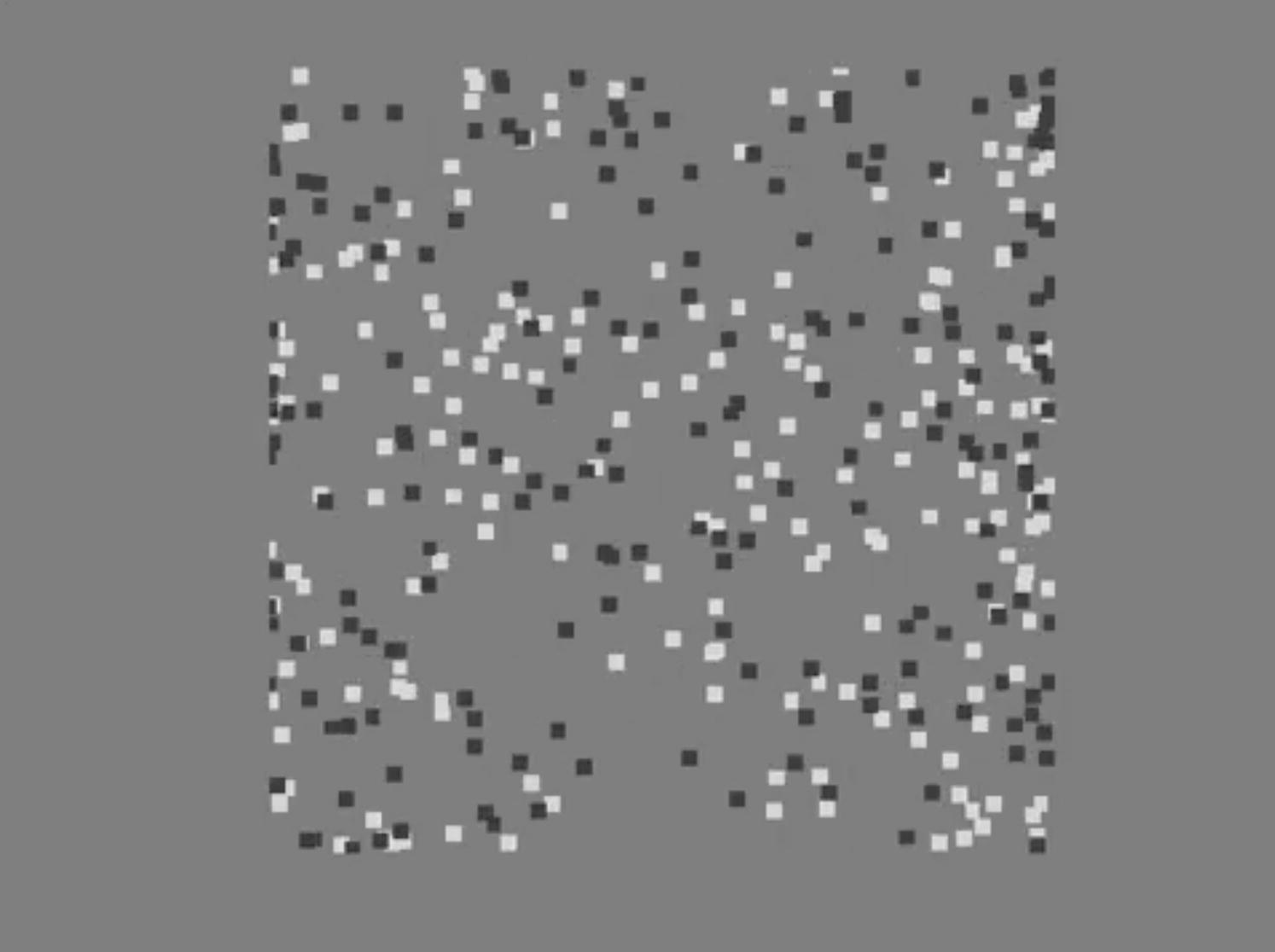

Supplement: Movie S1. Ambiguous, Rotating SFM Cylinder, Related to Figure 1 — Illustration of the type of stimulus used in this study. Consistent with an orthographic projection of a three-dimensional cylinder, the peak velocity of the stimulus dots was at the midline of cylinder image; i.e., the dot velocity increased as they approached the midline and then decreased as they moved toward the lateral edges of the cylinder. The cylinder stimulus depicted here is presented at zero binocular disparity, and therefore its direction of rotation is ambiguous. While the percept at a given time is that of coherent rotation, the perceived direction will switch at intervals. For the 2 s presentation in our study, the stimulus should not switch, but in roughly half of the trials, the stimulus would be perceived as rotating CW, and in the other half as CCW. The cylinder stimulus is disambiguated by application of different binocular disparities to leftward- and the rightward-moving dots. [file mmc2.jpg]
